# Up Regulation of *cystathione γ lyase* and Hydrogen Sulphide in the Myocardium Inhibits the Progression of Isoproterenol–Caffeine Induced Left Ventricular Hypertrophy in *Wistar Kyoto* Rats

**DOI:** 10.1371/journal.pone.0150137

**Published:** 2016-03-10

**Authors:** Ashfaq Ahmad, Munavvar A. Sattar, Hassaan A. Rathore, Mohammed H. Abdulla, Safia A. Khan, Maleeha Azam, Nor A. Abdullah, Edward J. Johns

**Affiliations:** 1 School of Pharmaceutical Sciences, UniversitiSains Malaysia, Penang, Malaysia; 2 Department of Physiology, University College Cork, Cork, Ireland; 3 Department of Biosciences, COMSATS Institute of Information Technology, Islamabad, Pakistan; 4 Department of Pharmacology, Faculty of Medicine, University of Malaya, Kuala Lumpur, Malaysia; Max-Delbrück Center for Molecular Medicine (MDC), GERMANY

## Abstract

Hydrogen sulphide (H_2_S) is an emerging molecule in many cardiovascular complications but its role in left ventricular hypertrophy (LVH) is unknown. The present study explored the effect of exogenous H_2_S administration in the regression of LVH by modulating oxidative stress, arterial stiffness and expression of *cystathione γ lyase* (CSE) in the myocardium. Animals were divided into four groups: Control, LVH, Control-H_2_S and LVH-H_2_S. LVH was induced by administering isoprenaline (5mg/kg, every 72 hours, S/C) and caffeine in drinking water (62mg/L) for 2 weeks. Intraperitoneal NaHS, 56μM/kg/day for 5 weeks, was given as an H_2_S donor. Myocardial expression of *Cystathione γ lyase* (CSE) mRNA was quantified using real time polymerase chain reaction (qPCR).There was a 3 fold reduction in the expression of myocardial CSE mRNA in LVH but it was up regulated by 7 and 4 fold in the Control-H_2_S and LVH-H_2_S myocardium, respectively. Systolic blood pressure, mean arterial pressure, pulse wave velocity were reduced (all P<0.05) in LVH-H_2_S when compared to the LVH group. Heart, LV weight, myocardial thickness were reduced while LV internal diameter was increased (all P<0.05) in the LVH-H_2_S when compared to the LVH group. Exogenous administration of H_2_S in LVH increased superoxide dismutase, glutathione and total antioxidant capacity but significantly reduced (all P<0.05) plasma malanodialdehyde in the LVH-H_2_S compared to the LVH group. The renal cortical blood perfusion increased by 40% in LVH-H_2_S as compared to the LVH group. Exogenous administration of H_2_S suppressed the progression of LVH which was associated with an up regulation of myocardial CSE mRNA/ H_2_S and a reduction in pulse wave velocity with a blunting of systemic hemodynamic. This CSE/H_2_S pathway exhibits an antihypertrophic role by antagonizing the hypertrophic actions of angiotensin II(Ang II) and noradrenaline (NA) but attenuates oxidative stress and improves pulse wave velocity which helps to suppress LVH. Exogenous administration of H_2_S augmented the reduced renal cortical blood perfusion in the LVH state.

## Introduction

Left ventricular hypertrophy (LVH) is a compensatory response of the heart against the elevated after load to maintain the ejection fraction. Persistent elevated hypertrophy progresses to dilation of the heart and decreased ejection fraction which leads to heart failure [1]. Frequently, the manifestation of ventricular hypertrophy is associated with a thickening of the myocardium, fibrosis [2] and altered gene expression[3]. Elevated blood pressure and LVH have been found to be surrogate markers of each other in many studies [4–6] and spontaneously hypertensive rats (SHR) have been used as model of LVH [7]. It has been reported that lowering blood pressure following administration of captopril resulted in a regression of LVH[4].

Hydrogen sulphide (H_2_S) is a well known gaseous transmitter having pleotropic effects on the cardiovascular system. Studies have shown that H_2_S can be produced from *cystathione γ lyase*(CSE) found predominately in the heart [8], cystathione β synthase (CBS) in mammalian tissue especially the kidney[9, 10] while 3-mercaptopyruvate sulphur transferase (MST) is responsible for H_2_S production in the brain[11]. H_2_S has been shown to attenuate cardiac hypertrophy induced by abdominal aortic coaractationin Sprague-Dawley rats [12]. A decreased concentration of H_2_S has a possible role in the pathogenesis of hypertension [13].Hydrogen sulphide (H_2_S) has been reported to modulate vascular tone [14]and has a cardio protective role [15]. Oxidative stress is considered to play a major contribution in the pathogenesis of LV remodelling [16–18]. H_2_S has an antioxidant role by suppressing NAPH oxidase [19], scavenging lipid peroxides [20, 21], potentiating catalase and superoxide dismutase while up regulating GSH [22] in the brain and endothelial cells which may play a therapeutic role in many diseased conditions where the suppression of oxidative stress is required.

As indicated above, exogenous H_2_S may be a therapeutic option for cardiovascular diseases including hypertension and cardiac hypertrophy while based on the recent literature reviews[23–25], the role of H_2_S in LVH has not yet been explored. So far what is unknown is the functional contribution of the H_2_S producing enzyme particularly CSE in LVH and the impact of up regulation of CSE on the heart following H_2_S administration on the regression of LVH. A recent report [26] has demonstrated that selective homocysteine lowering potently attenuates pressure overload-induced cardiomyopathy via reduced oxidative stress but highlighted the contribution of the *cystathione β synthase* (CBS) enzyme in pressure overload cardiac hypertrophy and heart failure.

Increased levels of vasoconstrictors, such as Ang II in a fructose fed model of LVH [27] and NA in isoprenaline/caffeine model have been reported [28–30]. However, the status of these vasoconstrictors in response to up regulation of the CSE/H_2_S pathway is unexplored.

Oxidative stress in cardiomyopathy and heart failure has been attributed to the selective homocysteine lowering which effectively attenuates pressure overload-induced cardiomyopathy via reduced oxidative stress [26]. However, the levels of conventional oxidative stress markers such as malanodialdehyde (MDA), superoxide dismuatse (SOD), glutathione (GSH) and total antioxidant capacity (T-AOC) inLVH and the contribution of H_2_S as antioxidant in the regression of LVH is also unknown. Increased oxidative stress is related to endothelial dysfunction while exogenous administration of antioxidants like vitamins C and E have been found to reduce the endothelial dysfunction by reducing pulse wave velocity and oxidative stress markers in essential hypertensive patients [31]. The present study hypothesized that there is a down regulation of CSE in the heart with an increased oxidative stress in the systemic circulation in LVH.We also hypothesized that exogenous administration of H_2_S would up regulate CSE in the heart by attenuating the LV index, heart index, thickness of myocardium while attenuating oxidative stress and endothelial dysfunction which would help suppress the progression of LVH.

## Materials and Methods

### Animals

Thirty six *Wistar-Kyoto* rats were obtained from the animal research and service centre (ARASC) at Universiti Sains Malaysia, Penang, Malaysia with an initial body weight ranging from 180 to 200g. The rats were acclimatized for 5 days in the transit animal room environment at the School of Pharmaceutical Sciences, USM and were supplied with food and water* ad libitum*. Animals were divided into two main groups; one for the cardiovascular investigations and the other for the molecular expression study of cardiac* cystathione γ lyase* (CSE mRNA). Each group comprised: Control, LVH, Control-H_2_S and LVH-H_2_S (n = 6 rats per group) for the acute studies while same groups for the cardiac CSE mRNAs expression studies in which each group consists of 3 animals and each animal has triplicate so total number of replicate were 9 in one group (n = 9). LVH was induced by the administration of 5 subcutaneous injections of isoprenaline of 5mg/kg at 72 h intervals with caffeine 62mg/L in the drinking water for a period of 2 weeks as previously reported [32, 33] but with minor modification as reported [30]. NaHS (H_2_S donor) was administered via an intra-peritoneal injection of NaHS (56μM) at the same time each day for 5 weeks [13]. Blood was collected from the tail vein and centrifuged at 5000rpm for 10 minutes; plasma was removed and stored at -70°C. After thawing, samples were taken for measurement of Ang II, NAand antioxidant markers. This protocol was approved by the Animal Research and Service Centre (ARASC) at Universiti Sains Malaysia with the approval number 2012/76/364.

### Relative Quantification of CSE mRNA Expression in the Heart of Control, LVH, Control-H_2_S and LVH-H_2_S Rats

#### Extraction of target tissue from animal

All equipment (scissors, blades, harvesting desk, beaker, tissue test tubes) was washed with RNAZap^®^ (Ambion, Life Technologies Corporation, USA) solution to maintain RNA integrity. Animals were killed by cervical dislocation. Hearts were harvested and immediately tissues were transferred into RNAlater^®^ solution (Ambion, Life Technologies Corporation, USA) at 4°C.

#### Disruption and homogenization of samples

TRIzole reagent (ambion, Life technologies, USA) was used to extract Total RNA according to the manufacturer’s guidelines. After the various sequential steps of homogenization of heart tissue, washing and elution, total RNA was extracted, optimized and quantified for purity and yield respectively using a microplate reader (Bio Tek Instrument. Inc., VT, USA). Total RNA was converted to cDNA using a High Capacity RNA-to-cDNA kit (Applied Biosystems, USA) according to instructions provided by manufacturer. Conversion of cDNA was done using particular settings for this procedure of Step One Plus RT-PCR (Applied Biosystems, Singapore).

TaqMan primers and probes for the CSE gene (Gen Bank accession No. NM_017074.1 and H_2_S Rn00567128_m1) were derived from TaqMan^®^-Gene Expression assays (Applied Biosystems, USA)[34]. Similarly, TaqMan primers and probes for the β-actingene (Gen Bank accession No. NM_031144.2 and Rn00667869_m1) were derived from TaqMan^®^-Gene Expression assays (Applied Biosystems, USA)[35, 36]. Quantitative RT-PCR reactions were carried out on 3 experimental animals of one group (3x4 = 12 animals) while each rat heart sample was further analyzed in triplicate. Amplification of the housekeeping enzyme (internal control) β-actin allowed sample loading and normalization to be determined. The relative quantification of the target gene CSE and internal control β-actin, was calculated using the comparative C_T_ (threshold cycle) method with arithmetic formula (2^-ΔΔCT^) [37].

### Measurement of H_2_S Concentration in Plasma and Myocardium of Control, LVH, Control-H_2_S and LVH-H_2_S Rats

A tail vein blood sample was taken on the final day and centrifuged at 5000rpm for 10 minutes [30]. The measurement of H_2_S in the heart tissue was followed as previously reported [38]. Heart tissue (50 mg) was homogenized in 0.5 ml of zinc acetate (1%) and mixed with 0.5 ml of borate buffer (pH 10.01). After this, a volume of 0.5 ml of N, N-2 dimethyl-p-phenylenediamine (20mM) and 0.5 ml of FeCL_3_ (300mM) were added to the tissue homogenate. Reaction tubes were immediately sealed and incubated for 30 minutes with shaking at 37°C. After incubation, all the samples were centrifuged and H_2_S concentration was measured using the same procedure as described for plasma H_2_S measurement method. This method has been used extensively to measure tissue H_2_S[13, 38–40].

### CSE Activity in Cardiac Tissue of Control, LVH, Control-H_2_S and LVH-H_2_S Groups

Cardiac tissue CSE activity was measured by a method [39, 41]. Briefly describing homogenate of myocardial tissue was suspended in 50 mmol/L ice cold potassium phosphate buffer (pH 6.8). The reaction mixture consists of 100 mmol/L of potassium phosphate buffer (pH 7.4), 10 mmol/L of L-cysteine, 2 mmol/L of pyridoxal 5-phosphate and 10% w/v of cardiac tissue. Cryo vial test tubes were used as centre wells and each containing 0.5ml of 1% zinc acetate to trap the gas. An Erlenmeyer Pyrex flask having volume of 25 ml was used to perform the reaction. Erlenmeyer Pyrex flask containing reaction mixture and centre wells were flushed with N_2_ and then sealed with paraffin film. The reaction was started by transferring the reaction flask from ice to shaking water bath at 37°C. A volume of 0.5 ml of 50% trichloroacetic acid was added to reaction mixture after incubation at 37°C for 90 minutes to stop the reaction. Flasks were sealed again and incubated at 37°C for 60 minutes to make sure the complete trapping of H_2_S released from the reaction mixture. All the content of centre wells were transferred to test tubes while each test tube containing 3.5 ml of water. Afterward, 0.5 ml of 20 mmol/L of N, N-2 dimethyl-p-phenylenediamine, sulphate in 7.2 mol/L of HCL was added, soon after followed by 0.4 ml of 30 mmol/L of FeCL_3_ in 1.2 mol/L HCL. Absorbance of the resultant reaction mixture was taken at 670nm. H_2_S concentration was measured by using standard curve of H_2_S solutions (3.125–100μM).

### Measurements of Noradrenaline in the Plasma of Control, LVH, Control-H_2_S and LVH-H_2_S Rats

Plasma NA levels were measured on day 35 as reported [30]. Measurement of plasma NA concentration required 50 μL of plasma by processed following the instructions and using ELISA kit purchased from LaborDiagnostika Nord GmbH & Co. KG, Nordhorn, Germany.

### Measurements of Angiotensin II in the Plasma of Control, LVH, Control-H_2_S and LVH-H_2_S Rats

Plasma Ang II levels were measured using a kit and following manufacturer’s manual. (Cloud-Clone Corp. Uscn Life Sciences Inc.). All reagents, samples and standards were prepared. Thereafter, 50μL of sample was added to each well followed immediately by 50μL of Detection Reagent A. The samples were well shaken, mixed and then incubated for 1 hour at 37°C. Aspiration and washing was repeated 3 times before 100μL prepared Detection Reagent B were added. The reaction mixture was incubated for 30 minutes at 37°C followed by 5 repetitions of aspiration and washing. A volume of 90μL Substrate Solution was added and incubated for 15–25 minutes at 37°C. After this step, 50μL of Stop Solution was added and the plate was read at 450 nm in the spectrophotometer.

### Measurements of Oxidant Stress Markers in the Plasma of Control, LVH, Control-H_2_S and LVH-H_2_S Rats

The oxidative stress markers, superoxide dismutase (SOD) malanodialdehyde (MDA), glutathione (GSH), total antioxidant capacity (T-AOC) and NO levels were measured in the plasma by using specialised laboratory kits (NJJC Bio Inc., Nanjing, China) following the instructions provided by the manufacturer.

### Measurement of Electrocardiogram in Anesthetized Rats of Control, LVH, Control-H_2_S and LVH-H_2_S Groups

Food was removed from the cages on the night before the experiment. The rats were anaesthetised with intraperitoneal pentobarbitone sodium (60mg/kg, Nembutal^®^, CEVA, France). Thereafter, a 3-lead surface ECG recording was taken using gold plated needle electrodes (ADInstrument, Sydney, Australia) inserted underneath the skin as previously reported [42, 43]. Recordings were done for 5 minutes using an amplifier attached to a data acquisition system (PowerLab, ADInstrument, Sydney, Australia). Data for each rat was taken as the average of 15 electrical impulses which were then averaged for each group of rats.

### Acute Experiment

After the completion of the ECG recording, the acute experiment was performed following previously reported procedures [30, 44, 45]. The trachea was exposed by a midline neck incision and a cannula inserted to provide ease of ventilation throughout the experiment. The right carotid artery was then cannulated (PP50, Portex, Kent, UK) and attached to a fluid-filled pressure transducer (model P23 ID Gould, Statham Instruments, UK) which was connected to a data acquisition system (PowerLab^®^, ADInstrument, Australia) to allow continuous monitoring of mean arterial blood pressure (MAP) and heart rate (HR). The left jugular vein was cannulated (PP50, Portex, Kent, UK) to permit administration of maintenance doses of anesthetic and saline infusion. A mid-line abdominal incision allowed exposure of the left kidney which was carefully covered with a saline soaked cotton pads to prevent drying. The iliac artery was cannulated (PP50, Portex, Kent, UK) and the cannula was attached to a second fluid-filled pressure transducer and connected to the PowerLab system for measurement of iliac mean blood pressure. A laser Doppler flow probe (ADInstruments, Australia) was positioned on the outermost layer of the cortex of the left kidney to record the renal cortical blood perfusion (RCBP). After completion of surgery, the rats were allowed to stabilize for 1 hour. The systemic haemodynamic data, MAP and HR and the RCBP were monitored continuously for 1 hour. The rats were euthanized at the end of the experiment with an overdose of anaesthetic. The pulse wave velocity (PWV) was measured by taking the propagation time from Power Lab data while propagation distance was measured manually by putting a thread from the insertion point of the carotid artery cannula to the insertion point of the iliac artery cannula [46, 47]. At the end of the acute experiment, the heart was harvested, dried and weighed to allow measurement of heart, LV and kidney indices. The atria, great vessels, and the right ventricle were snipped off along its septal insertion. The LV diameter wall thickness and LV internal volume were measured using vernier callipers as reported earlier[48].

### Histopathology of LV by Using PicroSirus Red Stain Kit

Samples of LV were also taken and preserved in 10% formalin. The histopathology preparation of the samples involved embedding, trimming and sectioning followed by staining of the heart tissue with PicroSirus Red (Polyscience, Inc. Germany)[30]. The procedure involved the use of three solutions, A, B and C. Firstly; the slides were dipped in solution A for 2 minutes then rinsed well in distilled water. Then the slides were placed in solution B for 60 minutes and then in solution C for 2 minutes. Thereafter, the slides were immersed in 70% ethanol for 45 seconds. Collagen in the LV tissue will give a red colour.

### Statistical Analysis

The statistical analysis for the study was undertaken using a one way analysis of variance (ANOVA) followed by a Boneferroni *post hoc* test using Graph Pad Prism (Graph Pad Software, San Diego California U.S.A) while gene expression data was analyzed by using Comparative method (ΔΔC_T_ method) StepOne^™^ Software, version 2.1 (Applied Biosystem, USA). All data are presented as mean ± SEM with significance at P<0.05.

## Results

### Relative Quantification of CSE mRNA Expression in Myocardium, Plasma and Tissue H2S Concentrations and CSE Activity in the Heart

**Fig 1:**
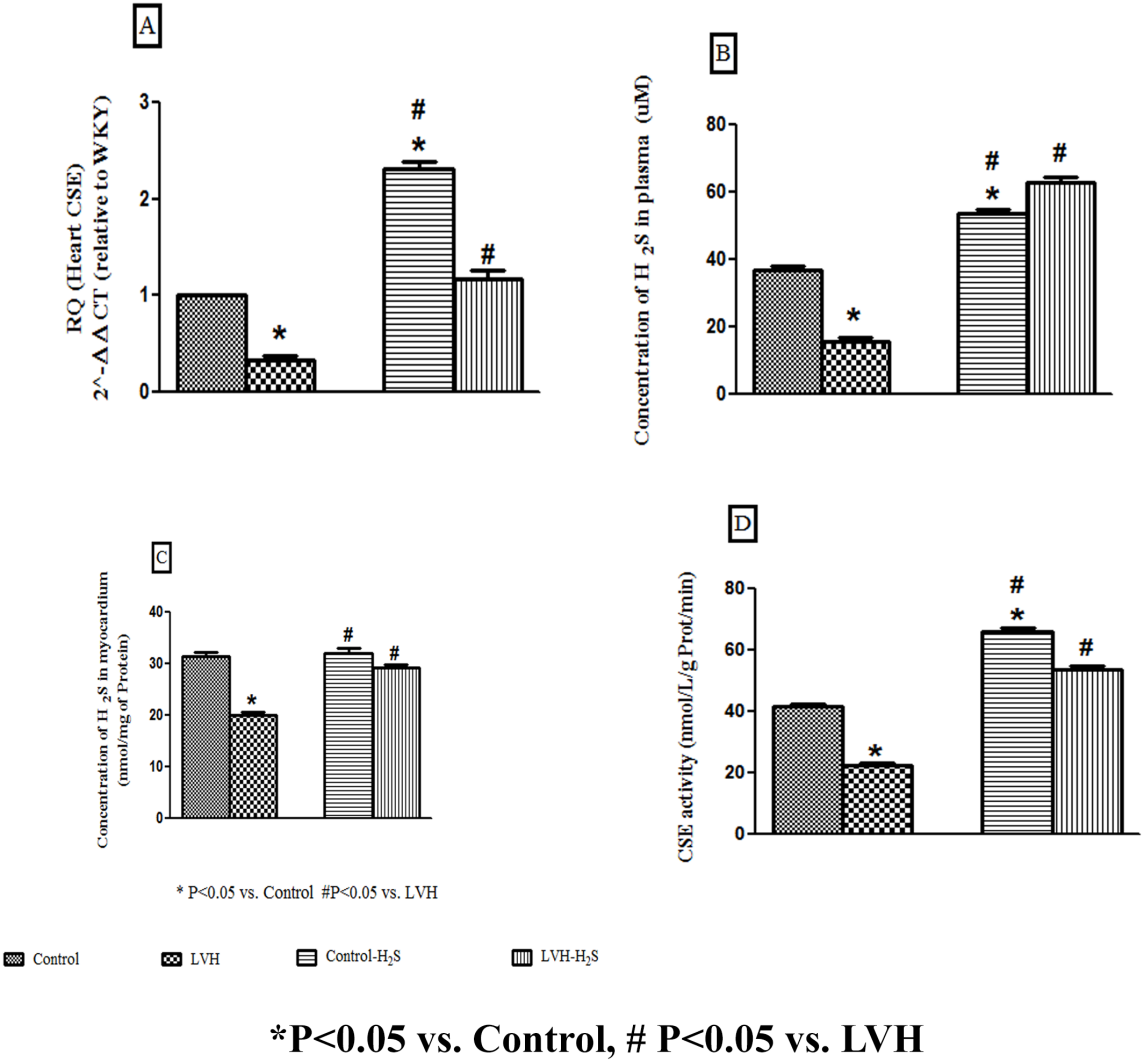
(A, B and C). Relative quantification of CSE mRNA expression in heart, concentration of H_2_S in the plasma and myocardium of Control, LVH, Control-H_2_S and LVH-H_2_S. All the data is expressed as mean± SEM. * P<0.05 represents comparison with control group while # represents comparison with LVH group.

It was observed that induction of LVH resulted in 3 fold decrease (CSE/β-actin mRNA ratio 0.32) in expression of CSE mRNA in the heart of the LVH as compared to the Control group (CSE/β-actin mRNA ratio taken as 1). Exogenous administration of H_2_S significantly up regulated (all P<0.05) the myocardial CSE mRNAs of Control-H_2_S and LVH-H_2_S when compared to the control group cardiac CSE mRNA as shown in Fig 1A.

Plasma hydrogen sulphide (H_2_S) in the LVH group of rats was significantly reduced (P<0.05) compared to H_2_S level in the Control group while exogenous administration of H_2_S significantly increased (all P<0.05) H_2_S plasma levels in Control-H_2_S and LVH-H_2_S (H_2_S (μM); Control: 37±1; LVH: 16±1; Control-H_2_S: 54±1 and LVH-H_2_S: 63±2) as shown in Fig 1B.

Myocardial hydrogen sulphide (H_2_S) levels in the LVH group of rats was significantly reduced (P<0.05) compared to H_2_S level in the Control group while exogenous administration of H_2_S significantly increased (all P<0.05) the myocardial H_2_S levels in Control-H_2_S and LVH-H_2_S (H_2_S (nmol/mg of protein); Control: 31±1; LVH: 20±1; Control-H_2_S: 32±1 and LVH-H_2_S: 29±1) as shown in Fig 1C.

CSE activity in the LVH group of rats was significantly reduced (P<0.05) compared to CSE activity in the Control group while exogenous administration of H_2_S significantly increased (all P<0.05) CSE activity in Control-H_2_S and LVH-H_2_S (CSE activity (nmol/L/g Prot/min); Control: 42±1; LVH: 23±1; Control-H_2_S: 66±1 and LVH-H_2_S: 54±1) as shown in Fig 1D.

### Plasma Concentrations of Noradrenaline and Angiotensin II

**Fig 2:**
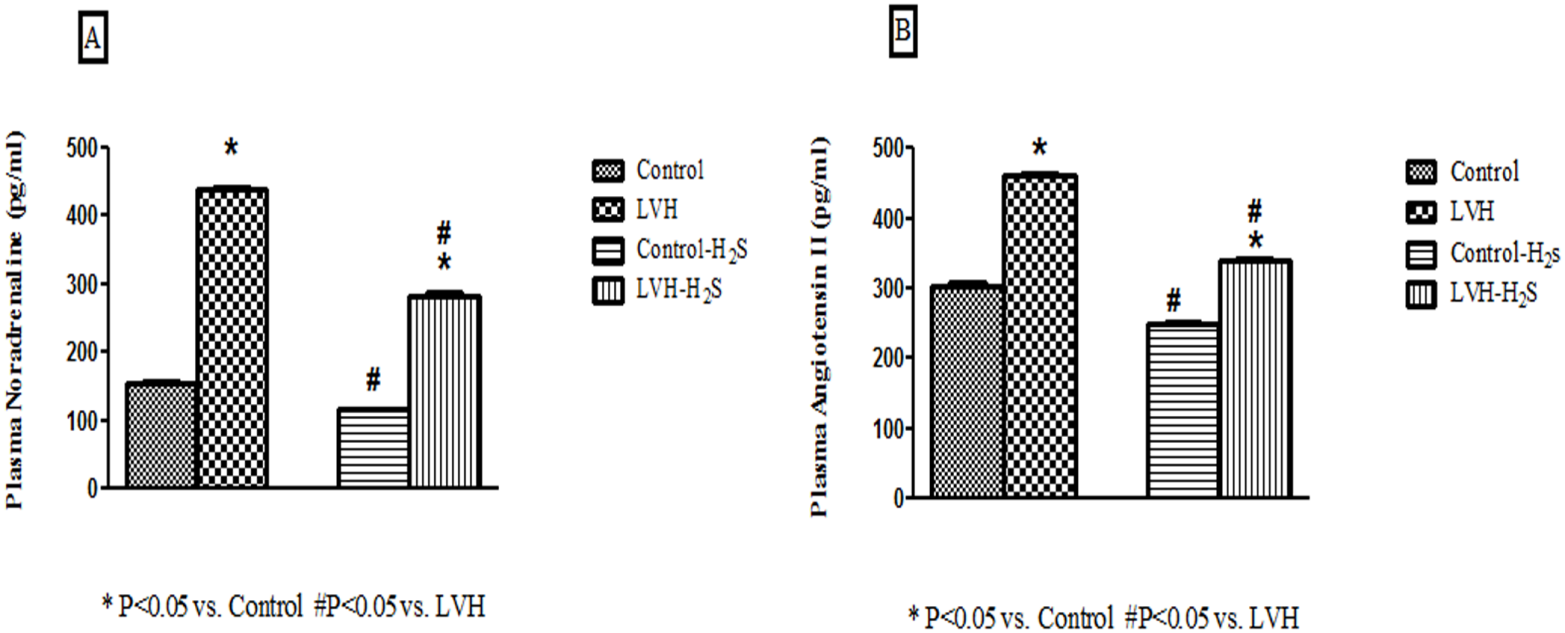
(A, B). Plasma concentration of noradrenaline and angiotensin II in plasma of Control, LVH, Control-H_2_S and LVH-H_2_S rat groups taken on day 35. All the data is expressed as mean± SEM. * P<0.05 represents comparison with control group while # represents comparison with LVH group.

In the LVH group, plasma NA levels were significantly elevated (P<0.05) compared to the Control group. Exogenous administration of H_2_S significantly reduced (P<0.05) the plasma NA levels in the LVH-H_2_S compared to the LVH group (NA in plasma (pg/ml; Control: 153±3; LVH: 438±4; Control-H_2_S:116±2 and LVH-H_2_S: 283±5) and these are shown are shown in Fig 2(A). Plasma Ang II levels were significantly elevated in the LVH (P<0.05) compared to the Control group. Exogenous administration of H_2_S reduced plasma Ang II levels in the LVH-H_2_S less than in the LVH group (Ang II in plasma (pg/ml; Control: 304±4; LVH: 460±3; Control-H_2_S:250±3 and LVH-H_2_S: 339±3) and are shown in Fig 2(B).

### Systemic Hemodynamics

The data showing the status of systemic hemodynamics in all groups is shown in Table 1. It was observed that induction of LVH resulted in significantly elevated (P<0.05) MAP compared to the Control group (Table 1). Exogenous administration of H_2_S significantly reduced (P<0.05) the MAP in the LVH-H_2_S group when compared to that of LVH group, whilst the Control-H_2_S group MAP was unchanged (MAP in mmHg: Control: 119±1; LVH: 142±5; Control-H_2_S:122±6 and LVH-H_2_S: 122±3; Table 1)). Renal cortical blood perfusion (RCBP) was significantly reduced (P<0.05) in LVH when compared to the Control group while exogenous administration of H_2_S significantly increased (all P<0.06) RCBP in Control-H_2_S and LVH-H_2_S to a level comparable to that of the Control group (RCBP in bpu; Control: 156±8; LVH: 94±6; Control-H_2_S:144±3 and LVH-H_2_S: 134±4; Table 1). Pulse wave velocity (PWV) was significantly reduced (P<0.05) in LVH compared to the Control group while exogenous administration of H_2_S significantly increased (all P<0.06) PWV in the Control-H_2_S and LVH-H_2_S groups to values comparable to those of the Control group (PWV in m/s; Control: 6±1; LVH: 8±1; Control-H_2_S:6±1 and LVH-H_2_S: 6±1; Table 1).

**Table 1:** MAP, RCBP and PWV of Control, LVH, Control-H_2_S and LVH-H_2_S are shown in Table 1. All the data is expressed as mean± SEM.

Parameters	Control	LVH	Control-H_2_S	LVH-H_2_S
MAP (mmHg)	119±1	142±5*	122±6#	122±3#
RCBP (bpu)	156±8	94±6*	144±3#	134±4#
PWV (m/s)	6±1	8±1*	6±1#	6±1#

MAP: mean arterial pressure; RCBP: renal cortical blood perfusion; bpu: blood perfusion unit; PWV: pulse wave velocity and m/s: meter per second

* P<0.05 represents comparison with control group while

^**#**^ P<0.05 represents comparison with LVH group.

### Cardiac Physical Indices

All data related to cardiac physical indices are shown in Table 2. It was evident that the heart index of the LVH group was significantly greater (P<0.05) than that of the Control group while exogenous administration of H_2_S to both Control and LVH groups significantly reduced (all P<0.05) the heart index in the LVH-H_2_S, but not Control-H_2_S group (heart index in %; Control: 0.26±0.0; LVH: 0.38±0.0; Control-H_2_S: 0.27±0.0and LVH-H_2_S: 0.34±0.0; Table 2).

**Table 2:** Heart index, LV index, thickness of myocardium and internal diameter of LV chamber of Control, LVH, Control-H_2_S and LVH-H_2_S are shown in Table 2. All the data is expressed as mean± SEM.

Parameters	Control	LVH	Control-H_2_S	LVH-H_2_S
Heart index (%)	0.26±0.0	0.38±0.0*	0.27±0.0#	0.34±0.0*#
LV index (%)	0.16±0.0	0.24±0.0*	0.19±0.0*#	0.21±0.0*#
Thickness of myocardium (mm)	1.6±0.0	3.3±0.0*	1.8±0.0*#	2.4±0.0*#
Internal diameter of LV chamber (mm)	5±0.0	3±0.0*	4±0.2*#	5±0.0#

mm: millimeter

* P<0.05 represents comparison with control group while

^**#**^ P<0.05 represents comparison with LVH group.

The LV index of the LVH group was significantly greater (P<0.05) than that of Control group while exogenous administration of H_2_S in both Control and LVH groups significantly reduced (P<0.05) the LV index in LVH-H_2_S, but not Control-H_2_S group (LV index in %; Control: 0.16±0.0; LVH: 0.24±0.0; Control-H_2_S: 0.19±0.0 and LVH-H_2_S: 0.21±0.0 Table 2).

The myocardial thickness in the LVH group was significantly greater (P<0.05) than that of the Control group while exogenous administration of H_2_S significantly reduced (all P<0.05) the thickness of myocardium in the LVH-H_2_S, but not Control-H_2_S (thickness of myocardium in mm; Control: 1.6±0.0; LVH: 3.3±0.0; Control-H_2_S: 1.8±0.0 and LVH-H_2_S: 2.4±0.0; Table 2).

The internal diameter of the LV chamber of the LVH group was significantly reduced (P<0.05) compared to that of the Control group and exogenous administration of H_2_S significantly increased (all P<0.05) the internal diameter of LV chamber in the LVH-H_2_S but not Control-H_2_S group (internal diameter of LV chamber in mm; Control: 5±0.0; LVH: 3±0.0; Control-H_2_S: 4±0.2 and LVH-H_2_S: 5±0.0; Table 2).

### Measurement of Electrocardiogram

All ECG data are shown in Table 3. The QRS complex of the LVH group was significantly increased (P<0.05) compared to the Control group while exogenous administration of H_2_S resulted in a significant decrease (P<0.05) in the QRS complex (QRS complex sec; Control: 0.018±0.0; LVH: 0.023±0.0; Control-H_2_S: 0.020±0.0 and LVH-H_2_S: 0.018 ±0.0 Table 3).

**Table 3:** QRS complex, R-R interval and R-amplitude of Control, LVH, Control-H_2_S and LVH-H_2_S. All the data is expressed as mean± SEM.

Parameters	Control	LVH	Control-H_2_S	LVH-H_2_S
QRS complex (Sec)	0.018±0.0	0.023±0.0	0.020±0.0	0.020±0.0#
R-R interval (Sec)	0.17±0.0	0.21±0.0	0.18±0.0#	0.18±0.0#
R-amplitude (mV)	0.53±0.0	0.74±0.0	0.54±0.0*#	0.63±0.0 #

Sec: second, mV: millivolts

* P<0.05 represents comparison with control group while

^#^ P<0.05 represents comparison with LVH group.

The R-R interval of the LVH group significantly greater (P<0.05) when compared to the Control group while exogenous administration of H_2_S resulted in significantly lower (P<0.05) in R-R interval compared to LVH group (R-R interval sec; Control: 0.17±0.0; LVH: 0.21±0.0; Control-H_2_S: 0.18±0.0 and LVH-H_2_S: 0.18±0.0; Table 3).

The R-amplitude in the LVH group was significantly higher (P<0.05) compared to that of the Control group andexogenous administration of H_2_S resulted in a significantly lower (P<0.05) R-amplitude compared to that of the LVH group (R-amplitude mV; Control: 0.53±0.0; LVH: 0.74±0.0; Control-H_2_S: 0.54±0.0 and LVH-H_2_S: 0.63±0.0; Table 3).

### Plasma Oxidative Stress Parameters

**Fig 3:**
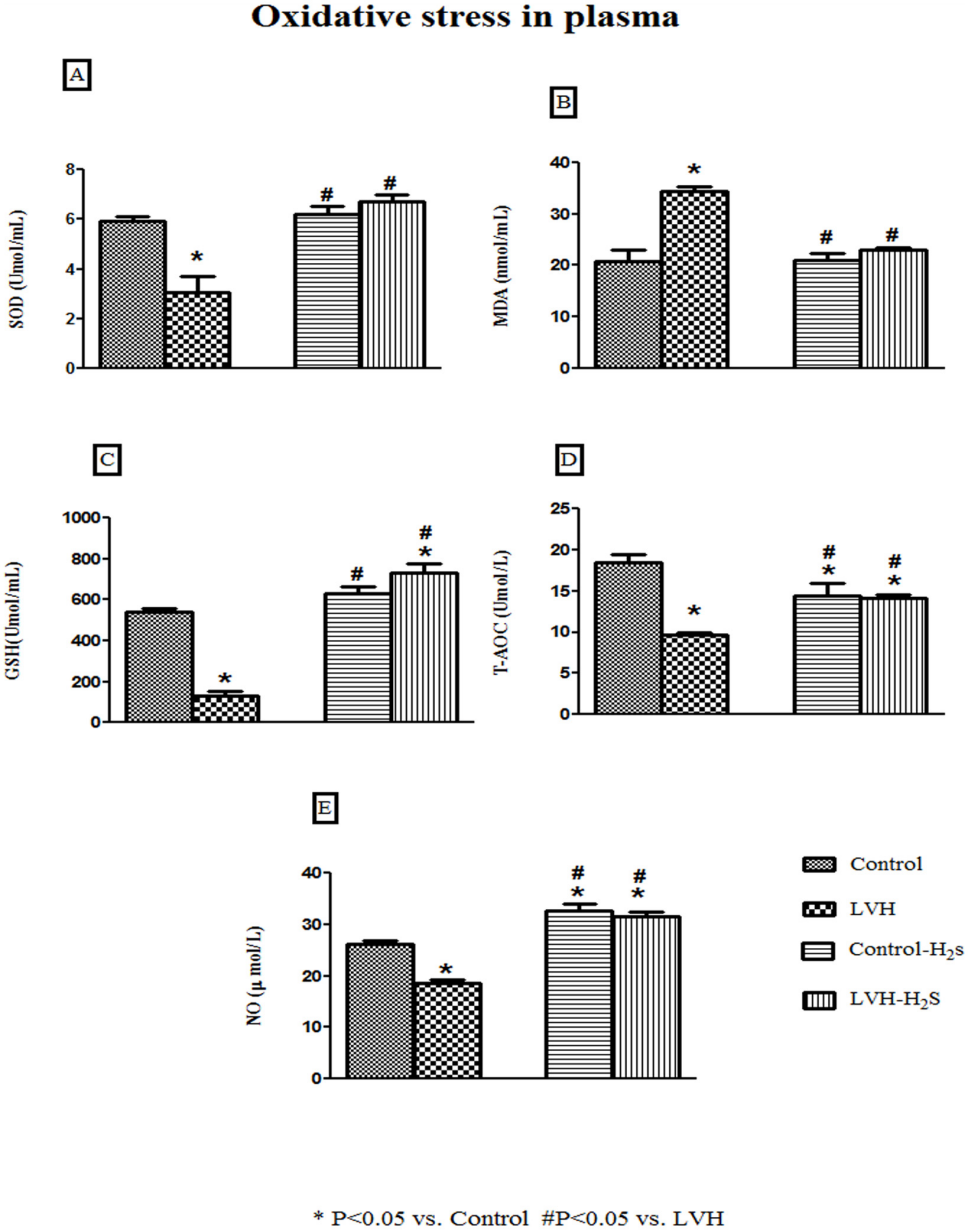
(A, B, C and D). Plasma concentration of superoxide dismutase (SOD), malanoaldehyde (MDA), glutathione (GSH) and total antioxidant capacity (T-AOC) of Control, LVH, Control-H_2_S and LVH-H_2_S rat groups taken on day 35. All the data is expressed as mean± SEM. * P<0.05 represents comparison with control group while # represents comparison with LVH group.

Plasma levels of superoxide dismutase (SOD) level in the LVH group rats was significantly lower (P<0.05) compared to those in the Control group and were significantly increased (all P<005) by the while exogenous administration of H_2_S in the LVH-H_2_S group (SOD (Umol/ml); Control: 6±0.2; LVH: 3±0.7; Control-H_2_S: 6±0.3 and LVH-H_2_S: 7±0.3) as shown in Fig 3A.

Plasma malanodialdehyde (MDA) was significantly higher (P<0.05) in the LVH group of rats compared to those of the Control group while exogenous administration of H_2_S significantly reduced (all P<0.05) MDA plasma levels in the LVH-H_2_S group (MDA (ɳmol/ml); Control: 21±2; LVH: 34±1; Control-H_2_S: 21±1 and LVH-H_2_S: 23±1) as shown in Fig 3B.

Glutathione (GSH) levels in plasma of the LVH group rats was significantly lower (P<0.05) compared to those of the Control group while exogenous administration of H_2_S significantly increased (all P<0.05) the GSH plasma levels in Control-H_2_S and LVH-H_2_S (GSH (Umol/ml); Control: 542±17; LVH: 134±19; Control-H_2_S: 629±37 and LVH-H_2_S: 731±45) as shown in Fig 3C.

Total antioxidant capacity (T-AOC) plasma levels of the LVH group of rats was significantly lower (P<0.05) compared to those of the Control group while exogenous administration of H_2_S significantly increased (all P<0.05) the plasma T-AOC levels in the LVH-H_2_S group but remained significantly (P<0.05) below that of the Control group (T-AOC (Umol/ml); Control: 18±1; LVH: 10±1; Control-H_2_S: 14±2 and LVH-H_2_S: 14±1) as shown in Fig 3D.

Plasma nitric oxide (NO) level in the LVH group of rats was significantly lower (P<0.05) compared to the plasma NO in the Control group while exogenous administration of H_2_S significantly increased (all P<0.05) the plasma NO in both Control-H_2_S and LVH-H_2_S (NO (μmol/ml); Control: 26±1; LVH: 19±1; Control-H_2_S: 33±1 and LVH-H_2_S: 32±1) as shown in Fig 3E.

### Histopathology of LV

**Fig 4:**
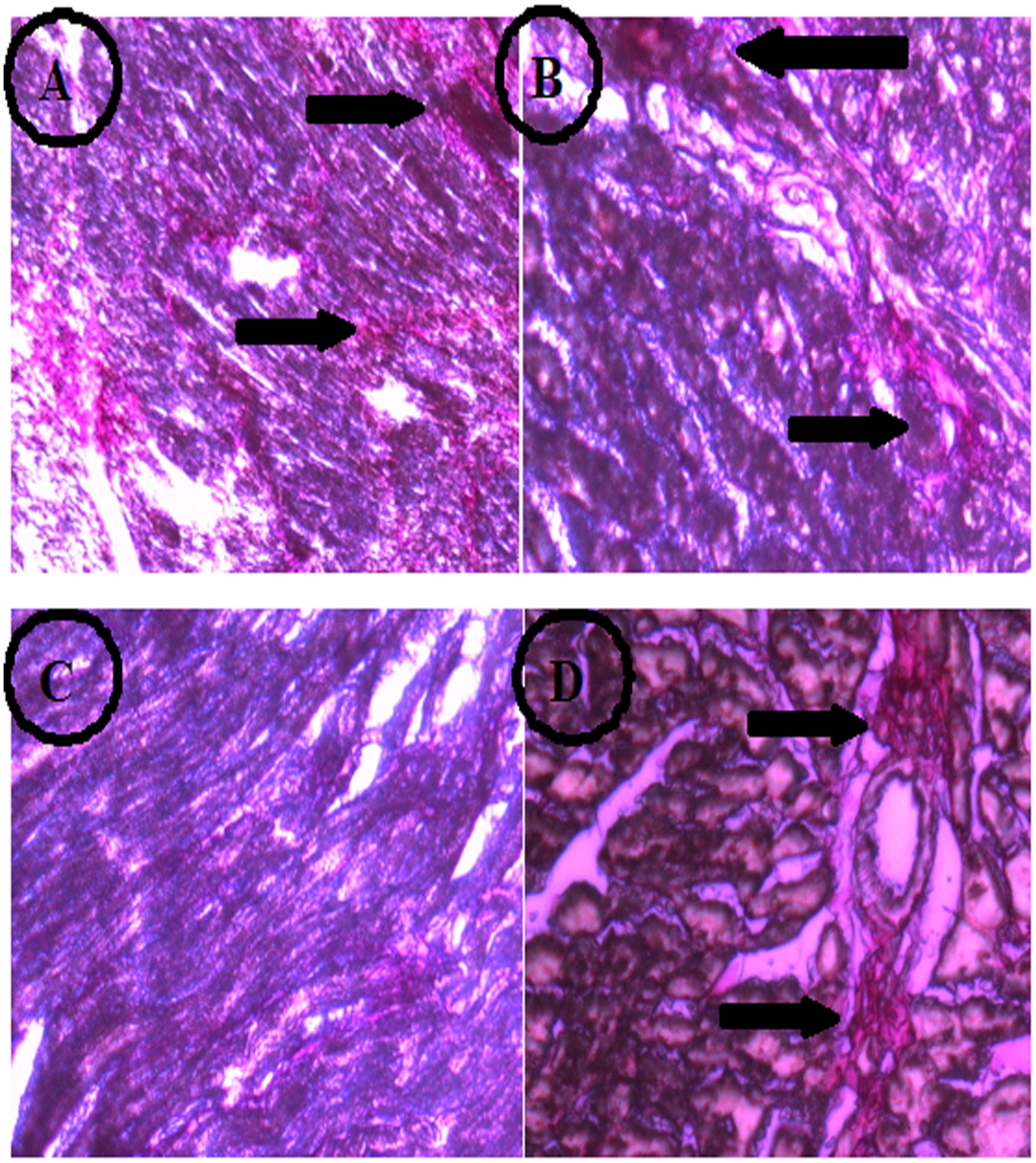
Deposition of collagen on the heart tissue of Control (A), LVH (B), Control-H_2_S (C) and LVH-H_2_S (D) rat groups taken on day 35 by using PicroSirius Red Staining. Arrows pointing out the deposition of collagen on myocardium in different groups.

PicroSirius Red Staining gave a red colour to the cardiac collagen which appears as thread-like structures. A normal amount of collagen is shown in the cardiac muscle of control rats which provides strength to muscle (Fig 4A). In the LVH group, as shown in Fig 4B, collagen appeared to be deposited as plaques which were present in different areas of the heart. Treatment with H_2_S in Control group, as shown in Fig 4C, shows normal thread like branches of collagen which are less dense than in the Control group. Exogenous administration of H_2_S in LVH rats, as shown in Fig 4D, reduced the collagen plaques which were not as dense compared to the LVH group. The slides show that collagen deposition in LVH is reduced after H_2_S administration.

## Discussion

The present study explored the hypothesis that in left ventricular hypertrophy there would be a down regulation of CSE mRNA in the heart with increased oxidative stress in the systemic circulation.We also hypothesized that exogenous administration of H_2_S would up regulate cardiac CSE associated with an attenuation of physical indices, as an antihypertophic agent on one hand while on the other hand blunt the oxidative stress and endothelial dysfunction thereby helping to suppress the progression of LVH. There were a number of novel observations. The first novel observation was that the induction of LVH resulted in the down regulation of CSE mRNA in the heart along with a corresponding decrease in H_2_S concentrations in the myocardium and plasma. Exogenous administration of H_2_S not only up regulated CSE mRNA expression in the heart but also suppressed the progression of left ventricular hypertrophy associated with a corresponding increase in H_2_S concentrations in the myocardium and plasma. The second novel finding was that down regulation of CSE resulted in increased LV index, heart index and thickness of myocardium and reduction in the internal diameter of the LV chamber. Up regulation of the CSE mRNA/H_2_S pathway in the heart resulting from exogenous administration of H_2_S highlighted an antihypertrophic role of the CSE mRNA/H_2_S pathway by attenuating the heart and LV indices, reducing the thickness of the myocardium and increasing the internal diameter of the LV chamber. The third novel observation was that the exogenous administration of H_2_S not only reduced the plasma concentrations of NA and Ang II but also antagonized the systemic actions produced by NA and Ang II. The fourth important finding was that the inhibition of the progression of LVH increased antioxidant mechanisms and improved end othelial dysfunction.

The present study demonstrated that there was a down regulation of CSE mRNA in the myocardium associated with the induction of LVH. This LVH could be due to isoproterenol as this is known to increase cardiac hypertrophy [49]. There may be other contributory factors causing LVH such as the elevated plasma levels of Ang II and NA in the LVH group which may be caused by a direct action isoproterenol acting on β-adrenoceptors in the heart to increase the force of contraction and at the kidney causing renin release from the granular cells [49]. Both Ang II and NA are also vasoconstrictors which are probably responsible for the elevated SBP and MAP in the present study. This increase in blood pressure and increased plasma levels of Ang II and NA may be the casual factors for the reduction in CSE mRNA and CSE activity in the heart and corresponding decrease in H_2_S concentrations in both plasma and heart. This view can be supported by the report that deletion of *cystathione γ lyase* is associated with the development of hypertension in mice [50] and H_2_S produced from CSE is known as a vasorelaxant and blood pressure regulator [51]. Reduction in CSE activity after administration of isoproterenol in present study is in line with the previous reported data [41].

Inhibition of CSE/H_2_S plays a significant role in the pathogenesis of hypertension [13] and the activation of this pathway could be a therapeutic objective for the treatment of hypertension and LVH. We have observed an upregulation of CSE mRNA and CSE activity in the heart and decrease in blood pressure in present study following activation of this pathway by exogenous administration of H_2_S. It was important to realise that up regulation of CSE mRNA in the myocardium of LVH-H_2_S resulted in reduced concentrations of Ang II and NA in the plasma. This can lead to the contention that up regulation of CSE mRNA may suppress the responses of Ang II and NA in LVH-H_2_S although these observations have not been made in past. The decrease in Ang II levels may be related to angiotensin converting enzyme inhibition by H_2_S [52] which would blunt Ang II production. However, the mechanisms underlying the decreased concentration of NA by H_2_S is unknown however one possibility may be a negative regulation of β-adrenergic receptors [53] by H_2_S which would lead to a reduction in cardiac contractility and a consequent reduction in the work of the heart and resolution of the LVH. This would be supported by the fact that β-blockers have been proven to be effective in causing regression of LVH [54].The protective effect of H_2_S in isoproterenol induced injury of themyocardium has also been reported [41]. This could arise due to the activation of potassium sensitive ATP channel by H_2_S as activation of these channels by diazoxide has resulted in regression of isoproterenol induced cardiac hypertrophy.[55]. We propose that the restoration of myocardial geometry by H_2_S might be due to up regulation of CSE in the as a result of activation of potassium sensitive ATP channels. It was evident from present study that collagen deposition was reduced in LVH following exogenous administration of H_2_S. Together, these observations would support the view that exogenous administration of H_2_S up regulated the CSE mRNA and increased CSE activity in the heart, reduced the plasma concentrations of Ang II and NA which lead to the reduction in blood pressure and inhibited the progression of LVH.

We observed in the present study that up regulation of myocardial CSE mRNA/ H_2_S reduced both heart and LV indices, thickness of the myocardium and increased the internal diameter of the LV chamber. As is evident from the present findings, plasma levels of Ang II and NA were not only elevated but also associated with the elevated physical indices of the heart and LV. This is consistent with previous reports that Ang II and NA are responsible for cardiac hypertrophy [12,56, 57]. The reduction in cardiac and LV indices following exogenous administration of H_2_S indicates an antihypertrophic role of the CSE/H_2_S pathway which may involve reducing the concentrations of Ang II and NA and antagonizing the growth promoting effects of Ang II and NA. These data are in line with other studies which reported the reduction in heart weight and heart index by H_2_S in the abdominal aortic coaractation (AAC) model [12]. However the disease induction model and species difference is an important difference in this latter study.

This attenuation in physical indices by H_2_S was also observed in pressure overload cardiomyopathy model following up regulation of the CBS enzyme [26]. The present study has emphasized the importance of the CSE enzyme which is another enzyme responsible for H_2_S production in myocardium in addition to CBS. One of the possible reasons for the antihypertrophic role of CSE/H_2_S is the decreased plasma concentration of Ang II which has been reported to be elevated in the AAC model and incriminated as a major culprit for hypertrophy [58]. The elevated Ang II in LVH is inhibited by H_2_S [52]. Another possible reason may be the vasodilator action of the H_2_S [59] which will reduce the after load on the heart, peripheral vascular resistance and ultimately attenuation of the stretch of the ventricular walls. A diuretic action of the H_2_S [60]might also be among the mechanisms leading to an unloading of the workload on the heart via a reduction of the volume loaded stress. Together, ACE inhibitor actions, negative regulation of β-adrenergic receptors, vasodilator and diuretic actions of the CSE/H2S pathway will not only reduce the heart, LV indices but also will result in reduction of the myocardium thickness and improve the internal diameter of LV chamber all of which are evident in the present study. This becomes one of the first studies reporting an antihypertrophic role of H_2_S and in which we explored how the up regulation of CSE mRNA/H_2_S promotes overcomes LVH by mitigating heart and LV indices, reducing the thickness of myocardium and increasing the internal diameter of LV chamber.

Cardiac hypertrophy develops in response to many stressors [61, 62] and the production of reactive oxygen species is evident in the findings of the present study. Oxidative stress plays a key role in cardiac and vascular abnormalities in different types of cardiovascular diseases which is why antioxidant therapy may be beneficial for combating these diseases [63]. So, the elevated plasma MDA, which is an enzymatic marker of oxidative stress, together with an attenuated level of SOD, an enzymatic marker of antioxidant activity, indicate elevated oxidative stress in the LVH model. This shows that there is an imbalance between pro-oxidants and antioxidant in LVH. Other antioxidant parameters, such as GSH and T-AOC are also attenuated indicating a dominant role for prooxidant mechanisms in LVH. The increased levels of Ang II may be one of the reasons underlying the increased oxidative stress [64] in the present model of LVH. Exogenous administration of H_2_S resulted in the attenuation of MDA levels, increased SOD, GSH and T-AOC in the plasma of the LVH model. These observations are in line with many previous studies supporting an antioxidant role for H_2_S in LV remodelling in different pathological conditions [65–68]. There are several reasons supporting notion and one of the best explanations for the antioxidant action of H_2_S is the scavenging ability of superoxide [67] and peroxynitrite[68]. Scavenging of these free radicals ultimately results in a reduction of MDA and improved SOD, T-AOC and GSH levels in the plasma. Another argument for improved antioxidant status could be due to the increased glutathione ability of H_2_S which suppresses the oxidative stress [61]. Improved antioxidant capacity in the present study is evident in LVH-H_2_S situation which mimics the antioxidant status of the body. This finding is similar to previous findings in lungs [69] where exogenous administration of H_2_S enhanced the T-AOC content. The antioxidant contribution of H_2_S in causing regression of LVH is also supported by a recently reported study [26] demonstrating up regulation of CBS in cardiomyopathy. Another possibility for the antioxidant action of H2S may be via its regulation of the NO pathway. NO is antioxidant on its own and inhibits xanthine oxidase and NADPH oxidase and maintains the normal O_2_^−^/NO homeostasis [70]. It is possible that following exogenous administration of H_2_S restores NO plasma availability in LVH in present study. A further antioxidant activity may reside in the ability of H_2_S to decrease the concentration of Ang II and plasma rennin activity [71]. It can be concluded that H_2_S increases the antioxidant status of the body by decreasing MDA, increasing SOD, GSH, T-AOC and NO in plasma by using its scavenging potential, increasing glutathione levels, regulating vascular NO and decreasing Ang II.

It became apparent that pulse wave velocity was significantly higher (33%) in the LVH group indicating a higher endothelial dysfunction as compared to the Control group. Interestingly vasoconstriction and elevated blood pressure are usually associated with greater arterial stiffness (endothelial dysfunction) which is a predictor of cardiovascular diseases [72]. Although isoprenaline produces a peripheral vasodilation but this action may be counteracted by an increased NA production induced by caffeine administration as observed in the present study. These elevated levels of NA and Ang II could cause an overall vasoconstriction and make the vasculature stiffer. Thus, the overall net effect is an increased left ventricular after-load and greater arterial stiffness ultimately leading to endothelial dysfunction.

Treatment with H_2_S resulted in decreased endothelial dysfunction compared to LVH and comparable to Control on day 35 which may be explained on the basis of multiple effects of H_2_S on blood vessels. It has been reported that the vasorelaxant effect of H_2_S [73, 74] may be due to its EDRF [59] and that a deficiency of H_2_S could ultimately lead to the pathogenesis of arterial hypertension [75].Furthermore, the endothelial dysfunction could be ameliorated by augmented vasorelaxation of blood vessels due to NO [76–78].

The antioxidant action of H_2_S may be expected to blunt the ROS degradation of NO and increase its availability in the vascular system. An increased NO availability following H_2_S has been reported earlier [73, 79]. The findings of our study are in line with these reports and is evidence of increased NO production in plasma after exogenous administration of H_2_S. In addition to this, increased oxidative stress as a result of LVH is evident in the study and results in vascular dysfunction [80, 81]. The Ca^++^ blocking ability of H_2_S [82] will result in vascular smooth muscle hyper polarization causing vasorelaxation [83] and is another mechanism by which H_2_S can reduce arterial stiffness. Ang II can cause arterial stiffness and ultimately LVH [84] but the ACE inhibitor like action of H_2_S can prevent this increased arterial stiffness. This could be another possible reason for H2S improving the arterial distensibility or stiffness. This reduced arterial stiffness can be attributed to H_2_S and can be considered as a therapeutic moiety for the regression of LVH.

Renal cortical blood perfusion (RCBP) was decreased by 36% in the LVH when compared to Control indicating compromised blood perfusion through kidney. Exogenous administration of H_2_S improved the RCBP by 40% when compared to LVH. This improvement in baseline RCBP due to exogenous administration of H_2_S can be explained in multiple ways. One of the possible reason is the role of H_2_S as an EDRF [59]. In present LVH model, the vasoconstriction produced by NA and Ang II are dominant factors mediating the reduced RCBP and the EDRF action of H_2_S may be expected to play a counter regulatory role to minimize the vasoconstriction in the kidney. This notion can be supported by other reports which demonstrated that exogenous administration of H_2_S resulted in greater pre-glomerulus arteriolar vasodilation, increased GFR and renal blood flow [38]. Local vasodilation by H_2_S in the kidney is likely because of the observed elevated plasma levels of H_2_S which suggest that there may be an up regulation of CSE mRNA and CSE activity throughout all tissues of the body. Another reason for increased RCBP in LVH-H_2_S is the ability of H_2_S to increase the NO production as discussed above. Another possible reason for improved RCBP is the antioxidant potential of H_2_S as decreased ROS production will reduce Ca^++^ influx mediated via sympathetic neurotransmission which will blunt NA secretion and consequently will result in an increase RCBP. This argument can be supported by an earlier report [85] which explains NA production due to enhanced ROS production. Thus, overall, decreasing ROS generation will reduce NA and Ang II production systemically leading to vasodilation and increases the RCBP.

## Conclusion

In summary, in this isoprenaline/caffeine model of LVH there is down regulation of the CSE/ H_2_S pathway in the heart with increased oxidative stress in the systemic circulation. Exogenous administration of H_2_S up regulated the cardiac CSE/ H_2_S pathway resulting in an attenuation of the increased LV index, heart index and thickness of the myocardium. This CSE/H_2_S pathway on one hand antagonizes the hypertrophic actions of angiotensin II and noradrenaline while on the other hand H_2_S attenuates the oxidative stress, endothelial dysfunction which together help to suppress the progression of LVH. Exogenous administration of H_2_S augmented the reduced renal cortical blood perfusion in LVH state.
